# Causal Relationship Between Blood Metabolites and Osteoporosis: A Two-Sample Mendelian Randomization and Genetic Correlation Analysis

**DOI:** 10.3390/bioengineering12050435

**Published:** 2025-04-22

**Authors:** Xu Liu, Guang Yang, Yusheng Li, Wenfeng Xiao, Bangbao Lu, Yaping Wang

**Affiliations:** 1 Department of Orthopedics, Xiangya Hospital, Central South University, Changsha 410008, China; 228112308@csu.edu.cn (X.L.); 228111092@csu.edu.cn (G.Y.); liyusheng@csu.edu.cn (Y.L.); xiaowenfeng@csu.edu.cn (W.X.); 2 National Clinical Research Center for Geriatric Disorders, Xiangya Hospital, Central South University, Changsha 410008, China; 3 Department of Geriatrics, Xiangya Hospital, Central South University, Changsha 410008, China

**Keywords:** metabolites, osteoporosis, Mendelian randomization

## Abstract

**Background:** Osteoporosis (OP) is a systemic bone disease often undiagnosed until fractures occur. Metabolites may influence OP, offering potential biomarkers or therapeutic targets. This study investigates the causal relationship between circulating metabolites and OP-related phenotypes using Mendelian Randomization (MR). **Methods:** GWAS data on 233 metabolic traits from 136,016 participants were analyzed through two-sample MR. Linkage disequilibrium score regression (LDCS) was used to estimate genetic correlations between metabolic traits and OP-related phenotypes, leveraging European ancestry linkage disequilibrium scores to account for polygenicity and stratification. MR employed the inverse-variance weighted (IVW) method, with sensitivity analyses via MR-Egger, MR-PRESSO, and weighted median methods to address pleiotropy and confounders. **Results:** LDCS identified significant genetic correlations between metabolites and bone mineral density (BMD) phenotypes, with total body BMD (toBMD) showing the strongest associations. Thirty-five metabolite traits, including apolipoprotein A-I, exhibited significant linkages. Among 79 metabolites influencing BMD, serum acetate levels were significantly associated with femoral neck BMD (OR: 1.28, 95% CI: 1.02–1.62), lumbar spine BMD (OR: 1.73, 95% CI: 1.32–2.27), and total body BMD (OR: 1.21, 95% CI: 1.04–1.42). Creatinine levels were consistently linked to reduced BMD, including lumbar spine BMD (OR: 0.88, 95% CI: 0.79–0.99). Triglycerides in IDL and VLDL particles also contributed to BMD variation. **Conclusions:** Significant genetic correlations and causal relationships were observed between specific metabolites and OP, highlighting key traits as potential biomarkers of bone health. These findings enhance the understanding of OP pathogenesis and suggest future preventive strategies.

## 1. Introduction

Osteoporosis (OP) is a systemic metabolic bone disease, primarily characterized by the deterioration of bone microarchitecture and decreased bone strength [1]. Known as the “silent disease”, OP often lacks noticeable symptoms in its early stages. Patients typically experience non-specific pain symptoms only in the later stages of the disease, with disease progression being generally slow [2]. The decline in bone mineral density (BMD) can continue for decades, and many patients become aware of their condition only after experiencing their first fracture [3]. The diagnosis of OP relies on specialized bone density testing techniques, such as dual-energy X-ray absorptiometry (DXA), which are often not included in routine physical exams, thus contributing to its low diagnosis rate [4]. Studies predict that by 2040, osteoporosis-related fractures in the United States will reach 3.2 million cases annually, with associated healthcare costs rising to over USD 95 billion per year, placing a significant burden on society [5].

Recent studies have identified multiple circulating metabolites associated with OP and low BMD, recommending their use as biomarkers to predict fracture risk in OP patients [6,7]. Moreover, recent research indicates a potential causal relationship between metabolites and OP, validated through several Mendelian randomization (MR) analyses, including those involving dehydroepiandrosterone sulfate and acetylcarnitine [8,9,10,11]. These causal inferences enhance our understanding of the role metabolites play in bone health, potentially offering novel biomarkers or therapeutic targets for the prevention and treatment of osteoporosis.

MR analysis is a method that uses genetic variants as instrumental variables (IVs) to explore causal relationships between exposures and outcomes [12]. The random allocation of alleles allows for effective control of unknown confounders, such as environmental factors [13]. However, we note that published MR studies on circulating metabolites as exposure factors have relatively small sample sizes, ranging from 1960 to 7824 individuals, and certain limitations exist in the genetic association analyses [8,9,10,11]. A recent genome-wide association study (GWAS) on circulating metabolites, published in *Nature*, included the largest sample size to date, with 136,016 individuals [14]. We aim to utilize MR analysis to explore the causal relationship between metabolites (apolipoproteins, cholesterol, triglycerides, albumin, various fatty acids, and other low molecular weight metabolites) and OP. The objective of this study is to enhance the reliability of the results by leveraging the larger sample size and addressing the limitations in previous genetic association analyses.

## 2. Methods

This study was conducted and reported following the STROBE-MR guidelines, which aim to enhance the reporting quality of observational studies utilizing Mendelian randomization.

### 2.1. Data Source

#### 2.1.1. Circulating Metabolic Biomarkers GWAS Data Sources

In this study, circulating metabolites were used as exposures for Mendelian randomization (MR) analysis [14]. Summary statistics from a large GWAS encompassing 233 circulating metabolic traits were obtained. This study involved 136,016 participants across 33 cohorts, with the majority being of European ancestry (6 Finnish and 21 non-Finnish cohorts) and six cohorts of Asian ancestry. All circulating metabolic biomarkers were quantified using nuclear magnetic resonance (NMR) spectroscopy via a metabolomics platform. This platform provided detailed data on lipoprotein subclasses, their lipid concentrations and compositions, including measures of apolipoproteins A-I (apoA-I) and B (apoB), cholesterol, triglycerides, albumin, a variety of fatty acids, and other low-molecular-weight metabolites. A total of 233 circulating metabolite GWAS data have been uploaded to the GWAS Catalog (https://www.ebi.ac.uk/gwas/home (accessed on 12 January 2025)) and assigned IDs ranging from GCST90301941 to GCST90302173.

#### 2.1.2. OP and BMD Outcome Datasets

Summary statistics from the GWAS were obtained from publicly available databases, which include the following four types of bone mineral density (BMD) data: femoral neck BMD (fnBMD), lumbar spine BMD (lsBMD), forearm BMD (foreBMD), and total body BMD (toBMD). Additionally, we obtained GWAS data on OP from the FinnGen consortium. The four BMD types are treated as continuous variables, while OP is considered a binary variable. For the BMD phenotypes, individuals carrying genetic variants with an odds ratio (OR) greater than 1 are at a lower risk of developing OP. Detailed information on GWAS datasets used in this study is provided in Table S1.

### 2.2. Statistics

#### 2.2.1. Linkage Disequilibrium Score Regression Analysis

Linkage disequilibrium score regression (LDSC) was employed to estimate genome-wide genetic correlations between exposure and outcome traits. Initially, single-trait LDSC was applied to calculate SNP (Single-nucleotide polymorphism) based heritability, mean χ^2^, genome inflation factor (λ_GC_), and intercept for each GWAS summary statistic. The values of λ_GC_ and the intercept were used to assess polygenicity and potential confounding due to population stratification or cryptic relatedness [15]. Pairwise LDSC was then conducted to estimate genetic correlations between circulating metabolic traits and OP-related phenotypes using pre-computed linkage disequilibrium scores from individuals of European ancestry from the 1000 Genomes Project Phase 3 (available at https://alkesgroup.broadinstitute.org/LDSCORE/ (accessed on 12 January 2025)). Adjusted *p*-values were obtained using the Benjamini-Hochberg procedure in R version 3.5.3, with statistical significance set at *p* < 0.05 for both genetic correlation and MR analyses.

#### 2.2.2. Two-Sample MR Analysis

**Figure 1:**
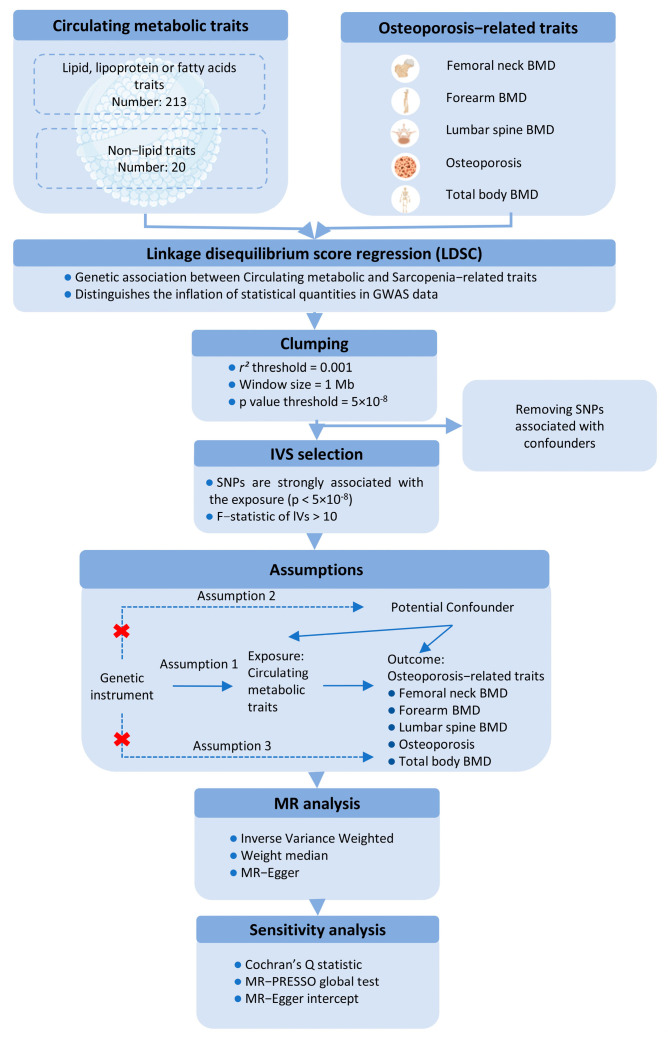
Schematic representation of the Mendelian Randomization (MR) framework used in this study, illustrating the three core assumptions required for causal inference between metabolites (exposure) and bone mineral density (BMD) outcomes.

This study utilizes a two-sample MR approach to investigate the causal effects of circulating metabolic traits on OP-related traits, using genetic variants as IVs. To ensure the robustness of the MR analysis, we adhered to three fundamental assumptions: (1) the IVs must be strongly associated with the exposures; (2) the IVs must be independent of any potential confounders influencing the exposure-outcome relationship; and (3) the IVs must not be directly associated with the outcomes, except through the exposures (Figure 1).

For the first assumption, SNPs with a *p* < 5 × 10^−8^ and an F-statistic > 10 were selected as IVs. A clumping procedure was applied (r^2^ > 0.001, clumping distance = 10,000 kb) to assess linkage disequilibrium (LD) between the selected SNPs. For the second assumption, SNPs associated with confounders such as age, sex, and BMI were excluded using PhenoScanner and PhenoScanner V2. For the third assumption, any SNPs significantly associated with the outcome phenotype (*p* < 5 × 10^−8^) were removed.

MR estimates were primarily calculated using the inverse-variance weighted (IVW) method with a random-effects model to account for potential heterogeneity. Sensitivity analyses included the weighted median method and MR-Egger regression, with the MR-Egger intercept test assessing directional pleiotropy (*p* < 0.05). Additionally, the MR-PRESSO test was applied to evaluate horizontal pleiotropy and identify outlier SNPs. Heterogeneity among the SNPs was assessed using Cochran’s Q statistic. A leave-one-out analysis was performed to determine whether any single SNP could disproportionately influence the IVW estimate. All MR analyses were conducted using the “TwoSampleMR” R package (version 0.6.4), with *p*-values adjusted using the Benjamini-Hochberg procedure in R (version 4.3.2). A *p*-value of <0.05 was considered statistically significant for both genetic correlations and MR analyses.

## 3. Results

### 3.1. LDSC Results

**Figure 2:**
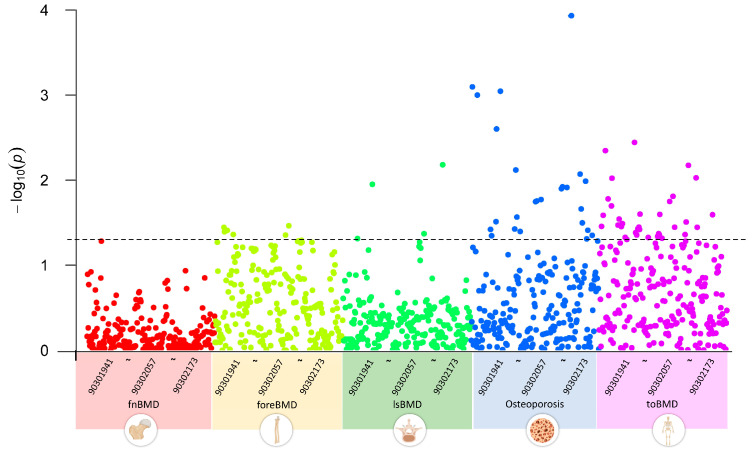
Genetic correlations between circulating metabolites and OP-related phenotypes using linkage disequilibrium score regression analysis, highlighting significant associations across BMD phenotypes for select metabolites. 903019041~90302173 represent the GWAS Catalog IDs of metabolites, incrementing from left to right. The plot data and description of the Catalog IDs are recorded in Table S2.

We first conducted a multivariate LDSC analysis using GWAS summary data to explore the genetic correlations between GWAS datasets of five OP-related phenotypes and 233 metabolites. The results indicated that, with a threshold of *p* < 0.05, none of the 233 circulating metabolite traits showed significant genetic associations with fnBMD. Five metabolite traits, including esterified cholesterol, were significantly associated with foreBMD. Four metabolite traits, including glycine, were significantly associated with lsBMD. Twenty-five metabolite traits, including alanine, were significantly associated with osteoporosis. Thirty-five metabolite traits, including apolipoprotein A-I, were significantly associated with toBMD (Figure 2). Table S2 provides a detailed overview of the circulating metabolite traits significantly associated with different OP-related phenotypes.

### 3.2. Causal MR Associations Between Metabolites and BMD

**Figure 3:**
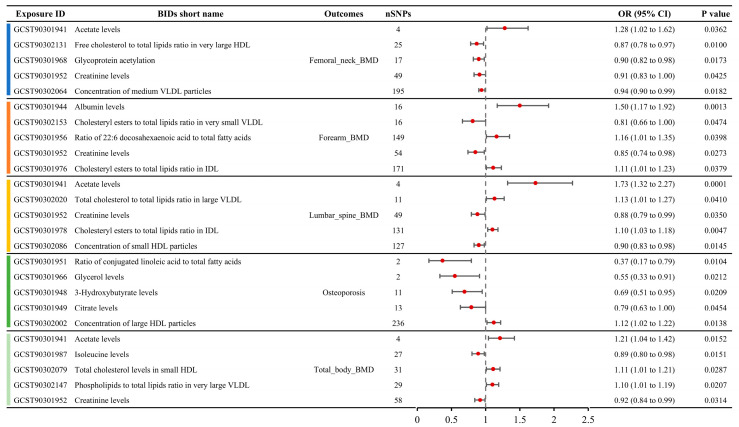
Causal relationships between metabolite concentrations and specific BMD outcomes obtained from MR analysis, showcasing the top five metabolites with the most significant causal effects on femoral neck, forearm, lumbar spine, and total body BMD.

A total of 79 metabolite traits showed causal relationships with BMD in the MR analysis, including femoral neck BMD, forearm BMD, lumbar spine BMD, and total body BMD (Table S3). Six metabolite traits were associated with femoral neck BMD, seven with forearm BMD, ten with lumbar spine BMD, and sixty with total body BMD. The MR results for the top five metabolite traits with the largest absolute effect sizes (OR-1) for the four BMD phenotypes are shown in Figure 3. For femoral neck BMD, the most influential metabolite trait was serum acetate levels (OR: 1.28, 95% CI: 1.02–1.62), followed by free cholesterol to total lipids ratio in very large high-density lipoprotein (HDL) (OR: 0.87, 95% CI: 0.78–0.97), glycoprotein acetylation (OR: 0.90, 95% CI: 0.82–0.98), creatinine levels (OR: 0.91, 95% CI: 0.83–1.00), and concentration of medium very low-density lipoprotein (VLDL) particles (OR: 0.94, 95% CI: 0.90–0.99). For forearm BMD, the top five metabolite traits, ranked by the absolute OR-1 value, were albumin levels (OR: 1.50, 95% CI: 1.17–1.92), cholesteryl esters to total lipids ratio in very small VLDL (OR: 0.81, 95% CI: 0.66–1.00), ratio of 22:6 docosahexaenoic acid to total fatty acids (OR: 1.16, 95% CI: 1.01–1.35), creatinine levels (OR: 0.85, 95% CI: 0.74–0.98), and cholesteryl esters to total lipids ratio in intermediate-density lipoprotein (IDL) (OR: 1.11, 95% CI: 1.01–1.23). For lumbar spine BMD, the top five metabolite traits ranked by absolute OR-1 value were acetate levels (OR: 1.73, 95% CI: 1.32–2.27), total cholesterol to total lipids ratio in large VLDL (OR: 1.13, 95% CI: 1.01–1.27), creatinine levels (OR: 0.88, 95% CI: 0.79–0.99), cholesteryl esters to total lipids ratio in IDL (OR: 1.10, 95% CI: 1.03–1.18), and concentration of small HDL particles (OR: 1.10, 95% CI: 1.03–1.18). For total body BMD, the top five metabolite traits were acetate levels (OR: 1.21, 95% CI: 1.04–1.42), isoleucine levels (OR: 0.89, 95% CI: 0.80–0.98), total cholesterol levels in small HDL (OR: 1.11, 95% CI: 1.01–1.21), phospholipids to total lipids ratio in very large VLDL (OR: 1.10, 95% CI: 1.01–1.19), and creatinine levels (OR: 0.92, 95% CI: 0.84–0.99).

### 3.3. Causal MR Associations Between Metabolites and OP

A total of seven metabolites showed causal relationships with the incidence of OP in MR analysis (Table S3). The top five metabolite traits ranked by absolute OR-1 value included the serum ratio of conjugated linoleic acid to total fatty acids (OR: 0.37, 95% CI: 0.17–0.79), glycerol levels (OR: 0.55, 95% CI: 0.33–0.91), 3-hydroxybutyrate levels (OR: 0.69, 95% CI: 0.51–0.95), citrate levels (OR: 0.79, 95% CI: 0.63–1.00), and concentration of large HDL particles (OR: 1.12, 95% CI: 1.02–1.22) (Figure 3).

### 3.4. Metabolites with Multi-MR Effects in Osteoporosis-Related Traits

**Figure 4:**
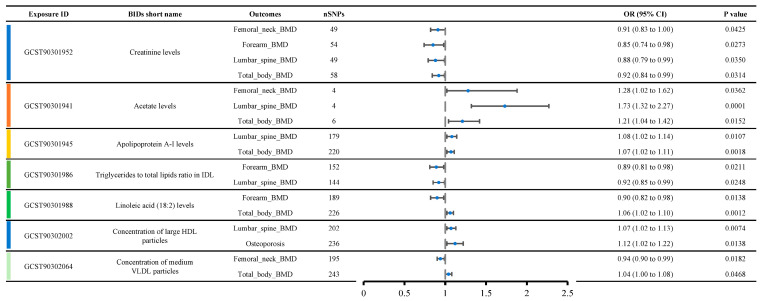
Metabolites show significant causal relationships with various OP-related phenotypes, emphasizing the consistency of creatinine and acetate across multiple phenotypes and thus underscoring their probable role in bone health.

Figure 4 shows a list of metabolite traits with significant causal relationships with various OP phenotypes. Creatinine levels were causally related to four OP-related phenotypes, including femoral neck BMD (OR: 0.91, 95% CI: 0.83–1.00), forearm BMD (OR: 0.85, 95% CI: 0.74–0.98), lumbar spine BMD (OR: 0.88, 95% CI: 0.79–0.99), and total body BMD (OR: 0.92, 95% CI: 0.84–0.99), suggesting a strong likelihood that creatinine levels contribute to reduced bone density. Acetate levels were causally related to three OP-related phenotypes, including femoral neck BMD (OR: 1.28, 95% CI: 1.02–1.62), lumbar spine BMD (OR: 1.73, 95% CI: 1.32–2.27), and total body BMD (OR: 1.21, 95% CI: 1.04–1.42), indicating that acetate levels are likely associated with increased bone density.

Additionally, we observed that Apolipoprotein A-I levels, the triglycerides to total lipids ratio in IDL, linoleic acid (18:2) levels, the concentration of large HDL particles, and the concentration of medium VLDL particles were each causally related to two OP-related phenotypes. Apolipoprotein A-I levels appeared to be associated with increased BMD, including lumbar spine BMD (OR: 1.08, 95% CI: 1.02–1.14) and total body BMD (OR: 1.07, 95% CI: 1.02–1.11). The triglycerides to total lipids ratio in IDL was likely associated with decreased BMD, including forearm BMD (OR: 0.89, 95% CI: 0.81–0.98) and lumbar spine BMD (OR: 0.92, 95% CI: 0.85–0.99). Interestingly, contradictory results were observed for linoleic acid (18:2) levels, concentration of large HDL particles, and concentration of medium VLDL particles. Linoleic acid (18:2) levels were associated with decreased forearm BMD (OR: 0.90, 95% CI: 0.82–0.98) but increased total body BMD (OR: 1.06, 95% CI: 1.02–1.10). The concentration of large HDL particles was associated with increased lumbar spine BMD (OR: 1.07, 95% CI: 1.02–1.13) but also with an increased incidence of osteoporosis (OR: 1.12, 95% CI: 1.02–1.22). The concentration of medium VLDL particles was associated with decreased femoral neck BMD (OR: 0.94, 95% CI: 0.90–0.99) but increased total body BMD (OR: 1.04, 95% CI: 1.00–1.08).

## 4. Discussion

OP is a metabolic disease characterized by an imbalance between bone formation and resorption, with osteoblasts and osteoclasts playing key roles in the bone remodeling process [16]. Previous studies have found that circulating metabolites, including amino acids and lipids, can promote or inhibit the bone remodeling process [17,18]. Researchers aim to identify BMD-related metabolic biomarkers to predict fracture risk and explore the potential causal relationships between circulating metabolites and OP, providing new insights into the biological mechanisms of OP. However, due to the influence of potential confounders, the findings have been inconsistent [18]. This study used a Mendelian Randomization design to naturally control for confounders and leveraged larger sample sizes to further enhance the reliability and statistical power of the conclusions.

This study first identified a strong causal relationship between creatinine levels and OP, specifically a negative correlation between creatinine levels and BMD. As early as 1993, Yendt et al. [19] found that women with primary OP had lower creatinine clearance rates, indicating that serum creatinine levels were higher in OP patients. However, Huh et al. [20] found that lower serum creatinine levels were independently associated with low BMD. Although we found that patients with chronic renal failure do experience reduced bone quality, this is due to decreased production of 1,25-dihydroxyvitamin D during renal insufficiency, leading to inadequate calcium absorption and, subsequently, secondary hyperparathyroidism. Secondary hyperparathyroidism increases the secretion of parathyroid hormone, which promotes bone resorption and releases calcium into the bloodstream, but this also results in decreased bone density [21]. Therefore, it appears that higher serum creatinine levels are associated with greater bone loss. However, there are currently no studies investigating how elevated creatinine levels directly affect BMD.

This study also found that acetate levels and apolipoprotein A-I levels were positively correlated with BMD, suggesting that both may serve as protective factors against OP. Acetate is an ester of acetic acid, and it can be produced through microbial fermentation in the gut as well as through fatty acid and ketone metabolism in the body [22]. Recent studies have shown that altering the gut microbiota in mice can increase fecal acetate levels, improve gut permeability, and inhibit osteoclastogenesis, thereby reducing bone loss [23]. Acetate’s downstream metabolite, acetyl-CoA, is a key substrate for the tricarboxylic acid cycle, providing energy for osteoblasts and osteoclasts [24]. Since osteogenesis requires significant energy to support osteoblasts, acetate levels may promote bone formation [24]. Acetyl-CoA is also a critical precursor for the synthesis of long-chain fatty acids. Studies have shown that in adipose (fat)-specific ACC1 knockout mice, skeletal growth was slower, trabecular bone density decreased, and proliferation of growth plate chondrocytes was reduced [25]. This indicates that the synthesis of long-chain fatty acids from acetyl-CoA is critical for bone formation, indirectly supporting the importance of acetate in bone quality. However, how acetate directly influences bone quality remains unclear [25]. Apolipoprotein A-I is the main structural protein of HDL, accounting for 70%, and plays a role in transporting cholesterol from peripheral tissues to HDL [26]. Animal studies have shown that apoA-I-deficient mice exhibit significantly reduced bone mass, with fewer osteoblasts and an increased number of adipocytes. Additionally, levels of chemokine CXCL12 and ANXA2 in their mesenchymal stem cells were significantly reduced [27]. However, a recent cross-sectional study by Sun et al. [28] found that higher levels of apolipoprotein A-I were associated with an increased risk of OP. Their findings seem to contradict our results, which indicate that apolipoprotein A-I is positively correlated with lumbar spine and total body BMD, acting as a protective factor against OP. In their study, the OP group included only 188 cases, while the non-OP group included 7555 cases [28]. We speculate that the positive association they observed may be due to the difference in sample size.

As the primary exposure factor in this study, lipoproteins can be classified into chylomicrons, HDL, IDL, low-density lipoprotein (LDL), and VLDL. Chylomicrons transport dietary fats, VLDL carries liver-synthesized triglycerides, LDL delivers cholesterol to cells (linked to heart risks), IDL bridges lipid metabolism, and HDL removes excess cholesterol, promoting heart health [29]. Their composition includes apolipoproteins and lipids, with the lipids consisting of triglycerides, cholesterol, and phospholipids [30]. The ratios between these lipids are constantly in dynamic flux [31]. The ratio of triglycerides to total lipids in IDL is the only lipoprotein exposure factor with a causal association to multiple outcomes. Additionally, we found that triglyceride levels in VLDL and IDL are negatively correlated with BMD. For LDL, triglyceride levels are positively correlated with toBMD, regardless of LDL particle size. Serum total triglyceride levels are also positively correlated with toBMD. However, some studies have found that higher total triglyceride levels are associated with an increased risk of developing OP [32]. We believe that the positive correlation between triglycerides and bone density may be due to fatty acid metabolism providing a significant amount of energy for osteoblasts [33]. Additionally, studies have shown that triglyceride metabolism in bone tissue is associated with osteoblast differentiation, suggesting that TG metabolism may play a supportive role in bone health [34]. The relationship between cholesterol and BMD is currently unclear. Some studies suggest that elevated cholesterol levels may reduce BMD, while others have found a positive correlation between cholesterol and BMD [35,36,37,38]. A study by Niu et al. [38] involving 440 participants reported that HDL-Cholesterol (HDL-C) levels in the blood were negatively correlated with bone density in the femur and femoral neck. A large multicenter study by Jiang et al. [35] also demonstrated that higher HDL-C levels are associated with an increased risk of osteopenia or osteoporosis. Our study also found that the ratio of free cholesterol to total lipids in very large HDL is negatively correlated with fnBMD, while total cholesterol in large HDL is positively correlated with the incidence of OP. However, we also noted that Xu et al. [37] found a positive correlation between HDL-C and BMD, as well as between HDL-C and 25(OH)D3 levels, the latter of which is known to promote bone formation. Our MR analysis also confirmed that free cholesterol in large HDL is positively correlated with lsBMD, and total cholesterol in HDL2 is positively correlated with toBMD. In our study, we found that cholesterol in HDL particles of different sizes exhibits causal relationships with BMD in opposite directions, which may explain the discrepancies among various studies.

Our study results differ significantly from previous research, primarily because the GWAS data used in this study focused on lipid metabolite traits, and various metabolites, including hormones and their derivatives, were not included in the analysis [14]. For example, dehydroepiandrosterone sulfate and androstenedione sulfate have been shown to be associated with OP in several previous studies, but they are not within the scope of this study [8,10,11]. The findings of Zhang et al. [7] also differ from ours, as their study reported a causal association between glycine, phosphatidylcholine, and BMD. However, our study found no causal relationship between serum glycine or phosphatidylcholine levels and OP or BMD phenotypes. In addition to glycine, valine and leucine have also been reported to be significantly associated with BMD, but in our study, neither valine nor leucine showed a causal relationship with OP or related phenotypes [18].

This study employed a two-sample MR analysis to explore the causal relationship between circulating metabolites and OP. Compared to previous MR studies, we utilized a recently published GWAS on circulating metabolites in *Nature*, which included the largest sample size to date. The study focused on five outcome measures, including forearm, femoral neck, lumbar spine BMD, osteoporosis incidence, and total body BMD, with an emphasis on the causal relationship between lipid metabolism and BMD, thus addressing certain gaps in the literature. However, our study also has some limitations. First, the exposure factors and BMD outcome datasets used in this study are primarily derived from European populations’ GWAS data, which limits the generalizability of the findings and overlooks the potential influence of genetic variation. Different populations may exhibit heterogeneity in their genetic structure and disease-associated risk loci. Second, the circulating metabolite traits discussed in this study are mostly lipid metabolism products, with other metabolites, such as amino acids and hormones, not included in the analysis. Third, the effect estimates derived from genetic correlation analyses represent estimates based on the current dataset and model. They cannot replace or be equated with effect estimates obtained from clinical observational studies. To obtain optimal clinical practice evidence, it is necessary to combine genetic correlation analyses with traditional epidemiological studies, real-world research, systematic reviews, or meta-analyses. Lastly, the GWAS data used in this study are based on multiple cohorts and lack detailed descriptions of population characteristics, which may lead to a certain degree of statistical bias.

## 5. Conclusions

The results indicate significant genetic correlations between two OP-related phenotypes, osteoporosis and toBMD, and circulating metabolites, revealing the complex interactions between circulating metabolites and osteoporosis. Additionally, our analysis identified multiple metabolite traits with causal relationships to OP-related phenotypes. We also found that seven metabolite traits, especially creatinine and acetate, have causal relationships with several OP-related phenotypes, suggesting that they may play important roles in the pathogenesis of OP. This study has significant implications for identifying individuals at risk for OP and for guiding dietary and lifestyle interventions to help maintain normal bone metabolism.

## Data Availability

The data are available upon request from the corresponding author. The datasets supporting the conclusions of this article are included within the article and its additional file.

## Supplementary Materials

The following supporting information can be downloaded at: https://www.mdpi.com/article/10.3390/bioengineering12050435/s1, Table S1: Information of included studies and consortia; Table S2: Description of single trait LDSC test results of 233 circulating metabolic biomarkers; Table S3: Summary MR analysis results for association between metabolite traits and OP and BMD.

## Abbreviations

OP	Osteoporosis
MR	Mendelian randomization
GWAS	Genome-wide association study
LDCS	Linkage disequilibrium score regression
IVW	inverse-variance weighted
BMD	Bone mineral density
DXA	Dual-energy X-ray absorptiometry
IVs	Instrumental variables
GWAS	Genome-wide association study
NMR	Nuclear magnetic resonance
apoA-I	Apolipoproteins A-I
apoB	Apolipoproteins B
fnBMD	Femoral neck BMD
lsBMD	Lumbar spine BMD
foreBMD	Forearm BMD
toBMD	Total body BMD
OR	Odds ratio
λ_GC_	Genome inflation factor
SNPs	Single nucleotide polymorphism
VLDL	Very low-density lipoprotein
IDL	Intermediate-density lipoprotein
HDL	High-density lipoprotein
LDL	low-density lipoprotein
HDL-C	HDL-Cholesterol
